# Molecular Determinants of Interactions between the N-Terminal Domain and the Transmembrane Core That Modulate hERG K^+^ Channel Gating

**DOI:** 10.1371/journal.pone.0024674

**Published:** 2011-09-15

**Authors:** Jorge Fernández-Trillo, Francisco Barros, Angeles Machín, Luis Carretero, Pedro Domínguez, Pilar de la Peña

**Affiliations:** Department of Biochemistry and Molecular Biology, University of Oviedo, Oviedo, Spain; Sackler Medical School, Tel Aviv University, Israel

## Abstract

A conserved *eag* domain in the cytoplasmic amino terminus of the human *ether-a-go-go*-related gene (hERG) potassium channel is critical for its slow deactivation gating. Introduction of gene fragments encoding the *eag* domain are able to restore normal deactivation properties of channels from which most of the amino terminus has been deleted, and also those lacking exclusively the *eag* domain or carrying a single point mutation in the initial residues of the N-terminus. Deactivation slowing in the presence of the recombinant domain is not observed with channels carrying a specific Y542C point mutation in the S4–S5 linker. On the other hand, mutations in some initial positions of the recombinant fragment also impair its ability to restore normal deactivation. Fluorescence resonance energy transfer (FRET) analysis of fluorophore-tagged proteins under total internal reflection fluorescence (TIRF) conditions revealed a substantial level of FRET between the introduced N-terminal *eag* fragments and the *eag* domain-deleted channels expressed at the membrane, but not between the recombinant *eag* domain and full-length channels with an intact amino terminus. The FRET signals were also minimized when the recombinant *eag* fragments carried single point mutations in the initial portion of their amino end, and when Y542C mutated channels were used. These data suggest that the restoration of normal deactivation gating by the N-terminal recombinant *eag* fragment is an intrinsic effect of this domain directed by the interaction of its N-terminal segment with the gating machinery, likely at the level of the S4–S5 linker.

## Introduction

Potassium channels encoded by the human *ether-a-go-go*-related gene (hERG) mediate the cardiac repolarizing current I_Kr_ [1], [2]. Mutations in the hERG gene and drug inhibition of hERG channels underlie inherited and acquired type 2 long QT syndrome [1], [3]–[8], a ventricular repolarization disorder that predisposes affected individuals to ventricular arrhythmia and sudden death [3], [9], [10]. Mammalian ERG channels also play an important role in setting the electrical behavior of various cell types, including pituitary lactotrophs, hippocampal astrocytes, glomus carotic body cells, pancreatic β-cells, smooth muscle myocytes, neurones and several tumour cells (reviewed in [11]–[15]).

The crucial determinant of the physiological roles of hERG is its ability to operate as an inward rectifier, even though the channel has the typical molecular topology of depolarization-activated channels [16]. Thus, due to the slow activation superposed on a fast inactivation upon depolarization, a voltage-dependent reduction in whole cell conductance and a bell-shaped I–V relationship is observed at positive voltages. During repolarization, hERG currents increase due to fast recovery from inactivation followed by a much slower deactivation. This maintains the channels open during longer periods of time at negative voltages, giving rise to the typical hERG tail currents [15]. In the case of the heart, this contributes to the repolarization of the cardiac action potential and to the prevention of arrhythmias induced by early after-depolarizations or ectopic beats [17], [18].

The molecular basis for the slow deactivation that contributes to the critical hERG tail currents is not totally understood. Previous work has indicated that an N-terminal domain conserved in the eag channel family comprising residues 1–135 of hERG (the *eag* domain), determines its slow deactivation, since channels with deletions of most of the amino terminus or of just the eag domain, with short deletions in the N-terminal most segment of the amino terminus, or with point mutations in the *eag* domain, all show rapid deactivation kinetics [19]–[26]. Since short deletions at the beginning of the N-terminus mimic the effect of more extensive amino terminal removal, it has been proposed that the initial subdomain segment of the *eag* domain (residues 1–26) acts as an essential regulator of hERG deactivation gating [22], [23], [26]. The crystal structure of the *eag* domain revealed the presence of a Per-Arnt-Sim (PAS) folding for residues 26–135, but it provided no structural information about the initial 1–26 N-terminal segment [21]. Recent NMR data identified two structurally differentiated regions in the N-terminal tail of hERG, an initial flexible and unstructured stretch extending up to residues 10–13, and an amphipathic α-helix constituted of amino acids 13–23 [26]–[28]. Subsequently, both the initial flexible segment and the alpha helical structure have been proposed to participate in conferring slow deactivation kinetics to the channel, but different interpretations about their contribution to this effect have been made. Thus, a critical role of the N-terminal α-helix and the PAS stabilizing the open state by an interaction with the C-terminal C-linker and cNBD domains [27] or with the S4–S5 linker in the central channel core [28], has been proposed. Alternatively, it has also been suggested that, rather than being involved in specific interactions with other domains, the amphipathic helix could act mainly as a spacer between the initial portion of the N-terminus and the PAS bound to some place in the central channel core, to adequately position the initial unstructured segment for interaction with the gating machinery at the level either of the S4–S5 linker or of the C-linker and cNBD domains [26].

We have recently demonstrated the physical proximity between the initial segment of the hERG N-terminus and the S4–S5 linker, since a disulfide bond can be formed between these intracellular domains carrying engineered cysteines, covalently locking the channels in a non-conductive state [29]. Such close proximity might allow for a physical interaction between these cytoplasmic domains. On the other hand, it has also been shown that the expression in *Xenopus* oocytes of a recombinant hERG N-terminal *eag* domain fragment tagged with a fluorescent protein, is able to restore normal deactivation gating properties of channels lacking almost the whole amino terminus [30], [31]. We have tried to gain some additional insights into the molecular requirements for this functional restoration to take place in transfected mammalian cells. In particular, we have checked the impact of structural alterations smaller than the truncation of the whole amino terminus in the channel molecule, since in addition to the well recognized influence of the initial *eag* domain structures on deactivation, both activation and deactivation gating can also be affected by the amino-terminal proximal domain under certain conditions [24], [25]. Therefore, the effect of the recombinant N1-135/YFP fragment was systematically tested against the Δ2-135 channel background that only lacks the portion of the protein corresponding to the exogenously added segment. We also checked the influence of single point mutations in the putatively interacting partners (i.e. the recombinant N-terminal domain, the N-terminal most segment of the channel N-terminus, and the S4–S5 linker), on re-establishment of normal slow deactivation. Our data suggest that the restoration of normal deactivation gating by the recombinant *eag* fragment is an intrinsic effect of this domain driven by the interaction of its initial segment with the gating machinery, likely at the level of the amino terminal portion of the S4–S5 linker.

## Materials and Methods

### Molecular Biology

The hERG, hERG Δ2-135 and hERG Δ2-370 clones have been previously described [24], [25]. To generate the single point mutants V3C, Y542C and G546C, site-directed mutagenesis was performed using the PCR-based overlap method with custom-made primers as described previously [24], [25], [29], [32]–[34]. For mammalian cell expression, the different hERG constructs and mutants were cloned into pcDNA3. Generation of the N- and C-terminally labeled WT hERG channels, TRH receptors labeled with CFP, and the hERG channel double labeled with both CFP and YFP, has been detailed elsewhere [35]. For C-terminal labeling of the hERG mutants, HindIII/BstEII or BstEII/BglII fragments from the different variants were used to replace the corresponding fragment in the full-length fluorescent hERG channel.

To generate the C-terminally labeled *eag* domain construct a HindIII/BamHI cDNA fragment containing the coding region corresponding to hERG residues 1–135 was cloned in-frame in pEYFP-N1 (Clontech). For this purpose a forward primer containing a HindIII site and the coding sequence corresponding to hERG amino acids 1–10 was used in PCR reactions with a reverse primer containing the coding sequence for residues 125–135 and the recognition site for BamHI. An analogous strategy was used to obtain the different variants with point mutations in the N-terminal segment of the labeled 1–135 YFP *eag* domain (V3C, R4C, G6C, V8C) by introducing the appropriate mutation in the forward primer used for amplification. All constructs were verified by standard fluorescence-based DNA sequencing to confirm the mutations and verify the absence of errors.

The membrane-localized CFP-YFP tandem construct (Rho-PYC) was kindly provided by Dr. Teresa Giráldez (University of La Laguna, Canary Islands, Spain). It contains the prenylation site of Rho added to the C-terminal domain of a YFP-CFP fusion [36], to anchor the fluorescent protein tandem to the lipids via the Rho-lipid binding motif.

### Tissue culture and transfections

Human embryonic kidney (HEK293) and Chinese hamster ovary (CHO) cells were grown at 37°C in a humidified atmosphere of 95% air and 5% CO_2_ and plated in 35 mm diameter tissue culture plastic dishes containing poly-L-lysine coated coverslips for electrophysiological measurements as previously described [35]. For TIRF and TIRF/FRET imaging, cells were seeded on 35 mm poly-D-lysine coated FluoroDish tissue culture dishes with cover glass bottom (WPI Inc, Sarasota, FL, USA). Cells were transiently transfected using Lipofectamine 2000 (Invitrogen) with 2–3 µg of plasmid DNA as previously described [35]. When co-transfecting constructs, plasmids were added in the indicated ratios, to maintain a proper molar ratio of expressed donor and acceptor fluorophores. When non-labeled channels were used for recordings, the plasmid DNA containing the channel construct was mixed with pEGFP-N3 encoding enhanced green fluorescent protein (eGFP) as a marker for transfection in a 5∶1 ratio. Recordings were typically performed 24–72 h after transfection.

### Electrophysiology

Ionic current recordings were performed at room temperature in the whole-cell configuration of the patch-clamp technique as detailed elsewhere [35]. The standard extracellular saline contained (in mM): 137 NaCl, 4 KCl, 1.8 CaCl_2_, 1 MgCl_2_, 10 glucose, and 10 HEPES (pH 7.4 with NaOH). The pipette solution contained (in mM): 140 KCl, 2 MgCl_2_, 0.7 CaCl_2_, 1.1 EGTA, and 10 HEPES (pH 7.4 with KOH). Kinetics parameters of activation and deactivation were obtained as previously described [24], [25], [34], [35], [37], [38]. Time constants of deactivation were determined from negative–amplitude biexponential fits to the decaying phase of the tail currents upon membrane repolarization at the indicated potentials, using a function y = *A_f_* exp(−*T*/*τ_f_*)+*A_s_* exp(−*T*/*τ_s_*)+C in which *T* is time, *τ_f_* and *τ_s_* are the time constants of fast and slow components, A_f_ and *A_s_* are the relative amplitudes of these components, and C is a constant. For simplicity, only the values of the deactivation time constants corresponding to the fast decaying current major component at negative voltages are shown on the figures.

### TIRF microscopy and FRET measurements

Through-the-objective TIRF microscopy was achieved with a Zeiss Axiovert 100 microscope equipped with a vibration isolation system (Newport, Irvine, CA, USA) to minimize drift and noise, a Zeiss 100× oil-immersion TIRF objective (1.45 NA; Oil, Alpha Plan Fluar), and a laser light delivery system consisting of an Argon-Ion LGK7880ML laser outputting 488 and 514 nm lines and a 442 nm Toptica iBeam 45 mW solid state laser. The excitation light was selected with an acoustic optical tunable filter (TILL Poly-line AOTF-controlled multi laser-line combiner; Till-Photonics, Gräfelfing, Germany). The light from a Polychrome IV monochromator was also combined with the laser into a single condenser (Till-Photonics). The Zeiss filter cube contained a polychroic mirror with reflection bands at 442 and 514 nm and band-passes at 475/30 and 560/60 nm (z442/514/633 rpc; Chroma Technology, Bockinham, VT, USA), and two z442/514× and z440/514 m excitation and emission filters (Chroma), respectively. CFP and YFP emissions were simultaneously collected using a DUAL-View Micro-Imager (Optical Insights, Santa Fe, NM, USA) equipped with a filter cube containing a HQ535/30 emission filter to isolate YFP emission, and a 505dcxr dichroic mirror for separation of CFP and YFP emission wavelengths. Images were collected and processed with an Imago 12-bit CCD camera (Till-Photonics). The camera, laser system and monochromator were controlled by TILLvisION 4.0 software, that was also used for image recording and processing. The TIRF angle was adjusted by eye to give the signature TIRF illumination to the experimental chamber.

FRET efficiency was measured by donor (CFP) de-quenching following photobleaching of the acceptor (YFP) fluorophore [35]. Due to the reduced depth of field of the 100×1.45 NA objective and to the critical dependence of the TIRF images on the focal plane, it was important to adjust the focus before every image acquisition. Whereas this was not relevant for the photobleachable YFP, it led to some unavoidable direct photobleaching of CFP that compromised the rigorous quantification of CFP fluorescence (F_CFP_) increases in a continuous way during the relatively long YFP photobleaching period. This also precluded the possibility of performing linear regression of F_CFP_ recovery versus F_YFP_ decrease to extrapolate the F_CFP_ values to zero F_YFP_ [35]. As an alternative, the objective was attached to a piezo focusing device controlled by the TILLvisION software, and used to acquire a *z* stack series of CFP fluorescence images both before and after YFP bleach. Election of the best in focus focused image of the series was subsequently performed either by eye or using the AutoFocus function of the software. Carefully adjusting the laser power to the necessary F_CFP_ output in each experiment, caused little CFP photobleaching (<2%). To attain a near maximal level of YFP bleach to accurately know the magnitude of the F_CFP_ increase, we used a combination of 514 nm laser and monochromator also tuned to 514 nm to permanently illuminate the YFP during the photobleaching period. As previously reported [36], this increased the bleaching level by ≈20%, up to around ≈95% of total YFP bleaching in 60–90 s. Individual cells in which the bleach level did not reach at least 90% were discarded from the analysis. Cells showing dim fluorescence leading to very low signal-to-noise ratios and those in which the F_YFP_/F_CFP_ ratio did not reach a ratio that ensured a proper YFP/CFP molar stoichiometry were also discarded [35]. Quantitative FRET levels were calculated by drawing regions of interest around the entire area of the cell and subtracting the background in a cell-free region for each image. Alternatively, the pre- and post-bleach cell masks were aligned using the TILLvisION software to compensate for any drift during the bleaching process, and images were compared pixel by pixel using NIH Image J [36]. Analogous results were obtained with and without this analysis variant. FRET was expressed as FRET efficiency (E_FRET_ in %), defined as described previously by the expressions E_FRET_ = E_FRET(APP)_ α_D_×100 = [1−(F_DA_/F_D_)] α_D_×100 in which F_DA_ and F_D_ are the fluorescence intensities of the donor before and after photobleaching of the acceptor, respectively, and α_D_ would indicate the fraction of total donor forming donor-acceptor complexes, a factor approaching 1 under our experimental conditions [35].

### Statistics

All values are presented as the mean ± S.E. Statistical significance was tested with the parametric unpaired two-tailed Student's *t*-test. When significant differences in standard deviation were present an alternate Welch's test or non-parametric Wilcoxon or Mann-Whitney test were also used. The results obtained were considered significant at *p*<0.05.

## Results

### Restoration of slow WT-type deactivation kinetics of N-terminus structurally altered hERG channels by a recombinant eag domain

**Figure 1:**
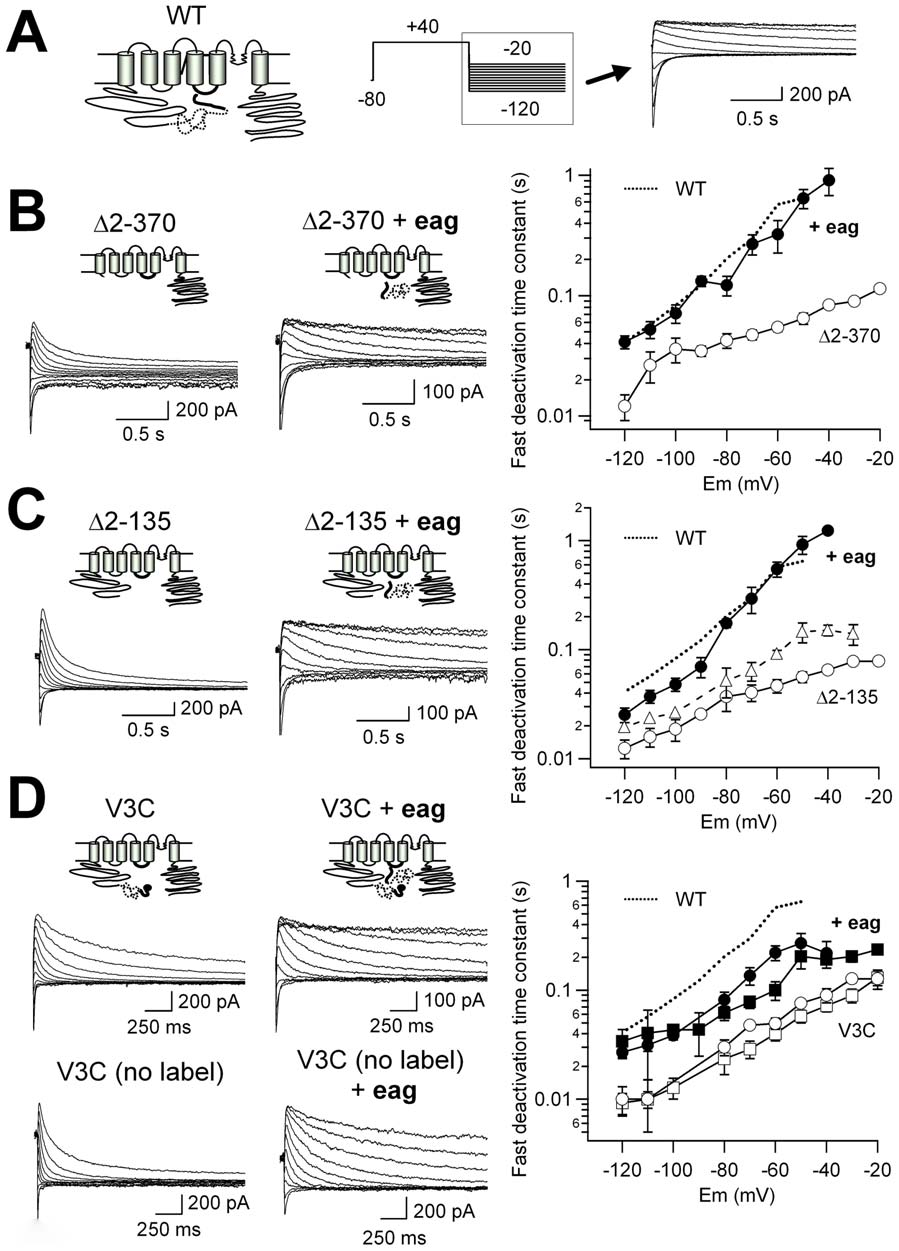
Recombinant *eag* domain-induced recovery of deactivation slowing in h-ERG channels carrying different structural alterations in the N-terminus. A. Schematic of the hERG. K^+^ channel with N-terminal *eag* domain and S4–S5 linker indicated. The PAS region of the *eag* domain is represented as a dotted line. The S4–S5 linker and the initial segment of the amino terminus corresponding to residues 1–26 are illustrated as thick solid lines. The pulse protocol used to study the deactivation kinetics and a family of representative tail currents obtained from WT channels at the times marked by the *dashed box*, are shown on the *right*. Note the initial increase in current due to recovery from inactivation and the subsequent decay from which deactivation rates are measured at every repolarization voltage. Note also the reversion of the current flow at around −90 mV. **B–D**. Effect of the N1-135/YFP recombinant fragment on deactivation kinetics of hERG channels truncated in the N-terminus (Δ2-370; panel B), lacking only the *eag* domain (Δ2-135; panel C) or carrying a single point V3C mutation (black sphere) at the beginning of the amino terminus (V3C, panel D). Channel schematics and families of representative tail currents elicited using the pulse protocols illustrated in panel A, are shown on the *left*. For clarity, the fluorescent labels located at the C-terminus of the channel constructs and the recombinant fragments are not represented in the schemes. Plots of fast deactivation time constants at different repolarization voltages derived from bi-exponential fits to the decaying phase of the tail currents (see Methods) are shown on the *right*. Data from WT hERG channels are also shown as *dotted lines* for comparison. Open and closed symbols correspond to data obtained in the absence or the presence of the recombinant fragment, respectively. DNAs for the channel and the N1-135/YFP fragment were maintained at a 3∶1 ratio during the transfection. The triangles in *C* illustrate deactivation rates from Δ2-135 channels co-expressed with the recombinant fragment using a DNA ratio of 10∶1. Circles and squares in the *right* panel of D represent the results of experiments using V3C channels with and without a C-terminal fluorescent label, respectively. *n*≥6 cells for each.

Truncation of almost the whole amino terminus of the hERG K^+^ channel induces a marked acceleration of deactivation kinetics [19]–[25]. To explore the structural determinants of this behavior, we used HEK-293 and CHO mammalian cells in which a recombinant hERG N-terminal *eag* domain fragment genetically fused to the fluorescent protein eYFP (N1-135/YFP) was coexpressed with N-terminus truncated channels (hERG Δ2-370), and also channels in which only the region corresponding to the *eag* domain was deleted (hERG Δ2-135). In both cases, channel constructs were also fused at the carboxy terminus to eCFP, since it has been demonstrated that fluorescent proteins fused to the hERG C terminus do not appreciably modify their gating properties [30], [35]. Apart from providing a suitable platform for subsequent spectroscopic analysis, the presence of the fluorescent label seemed to improve the functional expression level of some mutated channels (not shown), and did not influence the functional effects of the recombinant domains (see below). Consistent with previous results [30], [31], when N1-135/YFP was coexpressed with hERG Δ2-370 (N1-135/YFP: hERG Δ2-370 DNA ratio 1∶3), the deactivation kinetics were significantly (p<0.02 at all voltages) slowed to a rate similar to that of WT hERG (Fig. 1A,B). Almost identical results were obtained when the recombinant *eag* fragment was coexpressed with channels lacking only the protein segment corresponding to the *eag* domain (hERG Δ2-135, p<0.01), indicating that it is not necessary to remove the whole amino terminus, but only the segment used to restore the function (i.e. the *eag* domain), to achieve complete recovery of normal deactivation properties (Fig. 1C). Remarkably, the effect of the recombinant *eag* fragment was dose-dependent, since only a modest slowing of tail current decay was attained when the DNA ratio of N1-135/YFP to hERG Δ2-135 was lowered from 1∶3 to 1∶10 (Fig. 1C). On the other hand, further increases in the proportion of N1-135/YFP relative to hERG Δ2-135 channels (at DNA ratio 1∶1) did not modify the results obtained with the 1∶3 ratio (not shown). This saturation of the regulatory effect indicates that the deactivation slowing is not caused by a non specific binding of the recombinant domain to the channel molecule. It is important to note that the slowing down of closing by the recombinant *eag* domain is not due to a variation of the driving force at a given voltage, because the *I–V* relationship of the hERG Δ2-135 channel remained the same in the presence of N1-135/YFP (Fig. S1 and Table S1). Similar results were obtained with the hERG Δ2-370 channel (Table S1). Therefore, no differences in total potential energy driving deactivation (i.e., −(ΔG_o_-z_g_EF), see [25], ) were expected with or without the recombinant *eag* fragment. Note also that unlike the recovery in the closing rates observed with the recombinant *eag* domain, the rightward shifts in steady-state activation voltage dependence caused by the N-terminal deletions were not reversed with N1-135/YFP. This suggests that the effect of the recombinant *eag* fragment was selective for deactivation and that the shift in the equilibrium toward the closed state caused by the deletions was not corrected in the presence of the exogenously added *eag* domain.

It has been proposed that the initial segment of the *eag* domain constitutes an essential regulator of hERG deactivation [26]–[28]. We sought to use a single point mutant in the initial residues of the channel N-terminus, which had also shown fast rates of deactivation. Thus, we studied the behavior of a full-length channel construct carrying a Val to Cys mutation at residue 3 of the protein sequence (hERG V3C), both in the absence and the presence of the recombinant N1-135/YFP fragment. Surprisingly, the accelerated deactivation kinetics of the V3C channel mutant were significantly slowed by N1-135/YFP (Fig. 1D), despite the maintenance of a complete and structurally unaltered protein structure in the amino terminus (e.g. the amphipathic α-helix and the PAS domain). This demonstrates that the single point mutation is able to induce enough disengagement of the N-terminus from the closing machinery, allowing the recombinant fragment to bind and recover a substantial fraction of deactivation slowing.

We also checked if the presence of the fluorescent protein labels in both the channel and the recombinant fragment could influence the observed recovery of deactivation. However, almost the same result as that observed with the hERG V3C channel C-terminally labeled with CFP, was obtained with unlabeled hERG V3C (Fig. 1D). This indicates that the restoration of normal gating was due to a specific interaction of the recombinant *eag* fragment with the channel, and not to an unspecific effect directed by some residual oligomerization of the fluorescent labels.

### Role of the hERG S4–S5 linker in restoration of slow deactivation kinetics by the recombinant eag domain

**Figure 2:**
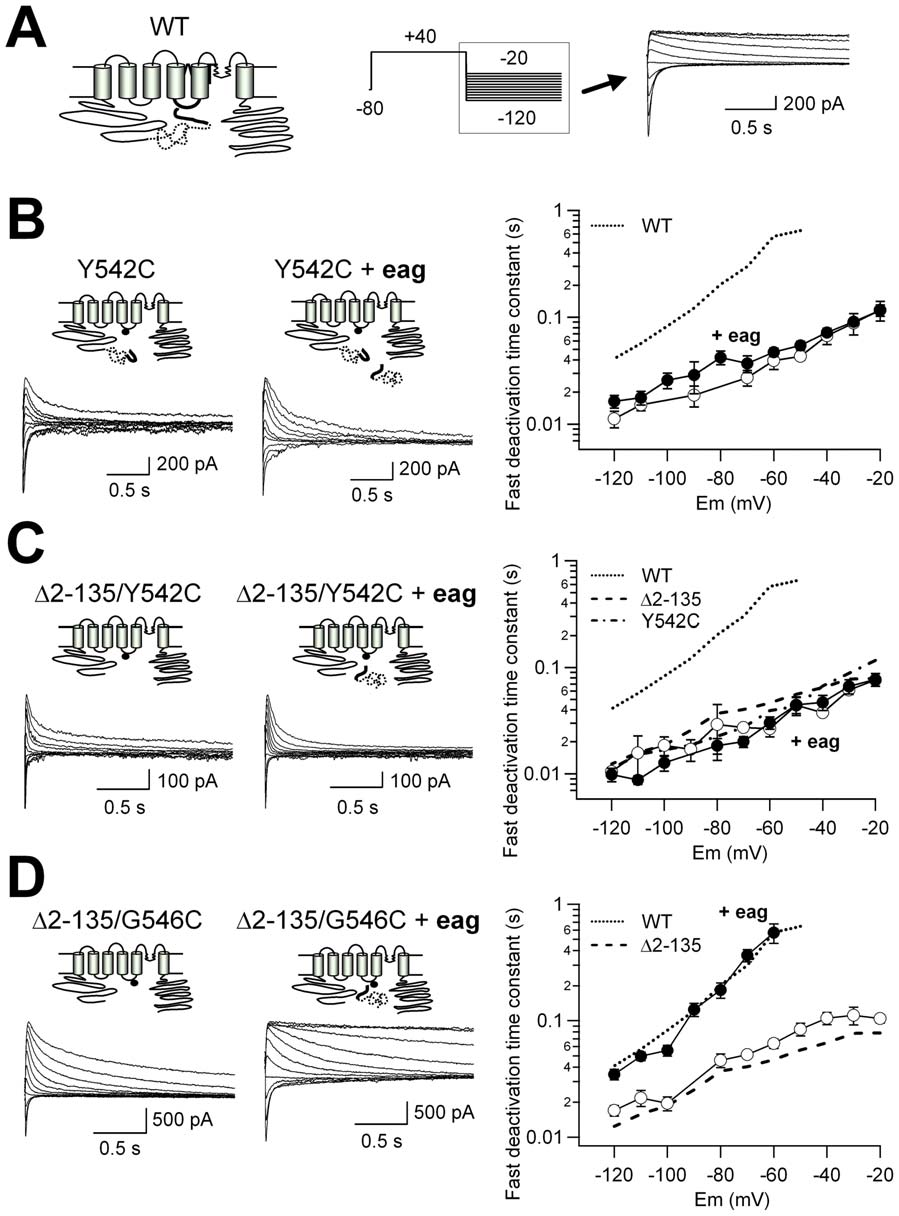
The recombinant N1-135/YFP fragment does not restore slow deactivation of hERG channels carrying a Y542C mutation in the S4–S5 linker. **A**. Schematic of the WT hERG K^+^ channel and representative tail currents obtained with the indicated protocol are shown for comparison. **B**. Lack of effect of the N1-135/YFP recombinant fragment on deactivation kinetics of Y542C channels. Channel schematics and families of representative tail currents are shown on the left. Black dots indicate the approximate position of the Y542C mutation. Plots of fast deactivation time constants at different repolarization voltages are shown on the *right*. Open and closed symbols correspond to data obtained in the absence or the presence of the recombinant fragment, respectively. Data from WT hERG channels are also shown as a *dotted line* for comparison. *n* = 10 and 6 for Y52C and Y542C+eag, respectively. **C**. The N1-135/YFP recombinant fragment does not slow down deactivation kinetics of Δ2-135 channels carrying a Y542C mutation in the S4–S5 linker. Open and closed symbols correspond to data obtained with the Δ2-135 plus Y542C construct in the absence and the presence of the recombinant fragment, respectively. Data from WT, Δ2-135 and Y542C hERG channels are also shown as *dotted* and *dashed lines* for comparison. *n* = 6 and 5 for Δ2-135/Y52C and Δ2-135/Y542C+eag, respectively. **D**. The presence of the N1-135/YFP recombinant fragment fully restores slow deactivation gating of Δ2-135 channels carrying the G546C mutation in the S4–S5 linker. Open and closed symbols correspond to data obtained with the Δ2-135 plus G546C construct in the absence or the presence of the recombinant fragment, respectively. Data from WT and Δ2-135 hERG channels are also shown as *dotted* and *dashed lines* for comparison. *n* = 6 and 8 for Δ2-135/G546C and Δ2-135/Y546C+eag, respectively.

Apart from its role coupling the voltage sensor to the activation gate [41], the S4–S5 linker region has been repeatedly proposed as a crucial structure for the influence of the N-terminus in the hERG gating properties [20]–[26], [28], [30], [40], [42]–[46]. In fact, it is well known that mutations in this linker result in accelerated deactivation rates similar to those observed when the amino terminal domain is deleted [19]–[26], [30], [41], [44], [45]. However, direct proof of a physical interaction between the amino terminal domains and the linker has been lacking. We reasoned that if the recombinant *eag* domain regulates deactivation through a direct interaction with the gating machinery at the level of the S4–S5 linker, structural perturbations in this linker could impair the effect of the exogenously added fragment. Consistent with this hypothesis, introduction of a Tyr to Cys mutation at residue 542 of the S4–S5 linker (hERG Y542C) accelerated channel deactivation to a level similar to that observed with the amino terminal modified channels. Furthermore, the presence of the mutation abolished the recovery of the closing kinetics induced by the N1-135/YFP fragment (Fig. 2A,B). An obvious interpretation of these results could be that the recombinant fragment is not able to compete with the wild-type *eag* domain, which occludes its access to the interaction site in the channel core. The results shown in Fig. 2C demonstrate that this is not the case. Thus, the presence of the recombinant N1-135/YFP domain also failed to recover the slow gating of the *eag* domain deleted channel that also carries a Y542C mutation (hERG Δ2-135/Y542C construct). Almost identical results were obtained with the Δ2-135/Y542C channel labeled with CFP at the C-terminus (not shown). This indicates again that the recombinant fragment effect was not driven by any unspecific interaction between the fluorescent protein labels.

It has been shown previously that introduction of a Gly to Cys mutation at residue 546 of the S4–S5 linker does not accelerate hERG channel closing [25], [46]. Also, a Cys at this position is not able to effectively establish a disulfide bond with a second cysteine located at the beginning of the hERG amino terminus [29]. To check the specificity of the Y542C effect, we generated a new channel construct (hERG Δ2-135/G546C) lacking the *eag* domain and with a G546C mutation in the S4–S5 linker. In contrast to the results observed with the Y542C mutation, in the presence of N1-135/YFP, the closing kinetics of the Δ2-135/G546C construct was slowed down to the level observed in wild-type hERG (Fig. 2D). These results further suggest that the structural modification of Tyr 542 at the beginning of the S4–S5 linker specifically impairs the noncovalent interaction of the soluble *eag* domain with the channel core, precluding the restoration of normal deactivation by the recombinant fragment.

### Impairment of the recombinant *eag* domain effect by mutations in the initial residues of its amino end

**Figure 3:**
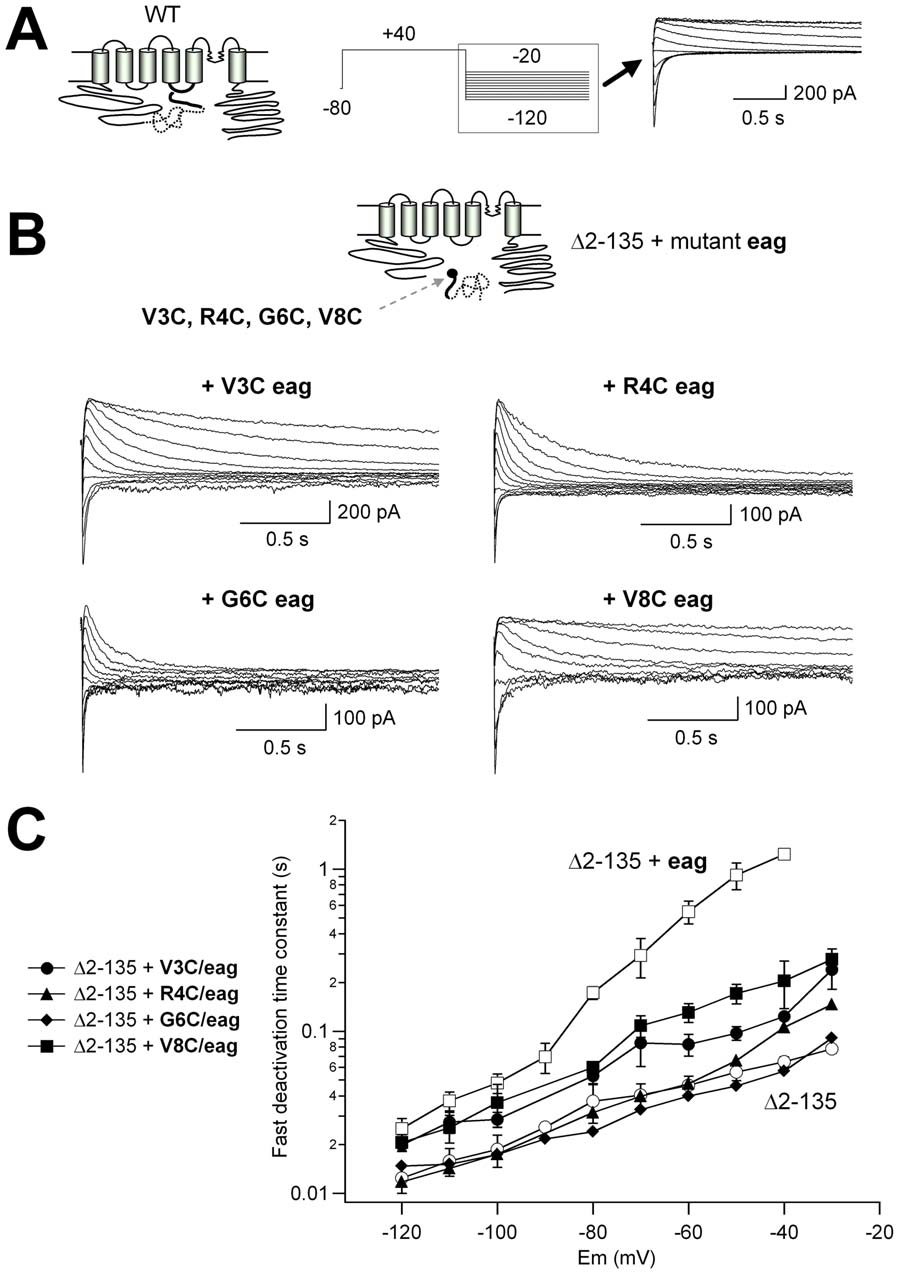
Mutations in the initial segment of the recombinant N1-135/YFP fragment affect its ability to recover deactivation slowing in hERG Δ2-135 channels. **A**. Schematic of the WT hERG K^+^ channel and representative tail currents obtained with the indicated protocol are shown for comparison. **B**. Schematic of the Δ2-135 channel and the recombinant fragment with the approximate positioning of the single point mutations (*black dot*). For clarity, the fluorescent labels located at the C-terminus of the channel constructs and the recombinant fragments are not represented in the schemes. **C**. Representative tail currents from CFP-labeled Δ2-135 channels co-expressed with the N1-135/YFP recombinant fragments carrying the indicated mutations. Horizontal scale bars correspond to 0.5 s. **D**. Plots of fast deactivation time constants at different repolarization voltages. Data from Δ2-135 channels with and without the recombinant fragment as in Figure 1 (*open symbols*) are also shown for comparison.

Recent results from our laboratory indicated that a disulfide bond can be formed between the N-terminal most segment of the amino terminus and the initial segment of the S4–S5 linker in which pairs of engineered cysteines have been introduced, demonstrating a physical proximity between these regions which may allow for a physical interaction between these cytoplasmic domains [29]. This prompted us to check whether mutation of the residues located at the beginning of the N1-135/YFP N-terminus could phenocopy the effect of the Y542C mutation. The effect of the recombinant fragment restoring normal deactivation kinetics of the hERG Δ2-135 channel was partially reversed when the recombinant N1-135/YFP carried the V3C mutation at the beginning of the amino terminus (Fig. 3). However, when this construct was coexpressed with the recombinant *eag* domain mutated at residues 4 or 6 of the protein sequence (R4C and G6C *eag* domain mutants), the deactivation kinetics were the same as those observed with the hERG Δ2-135 channel alone. Finally, the ability of the recombinant fragment to restore slower closing was significantly improved when the hERG Δ2-135 channel was coexpressed with a recombinant N1-135/YFP domain with a V8C mutation, indicating a certain degree of specificity for the mutation-induced impairment of the *eag* domain restoration effects. Since these data were obtained with Δ2-135 channels and N1-135 fragments both carrying fluorescent labels, they further demonstrate that the presence of the labels does not act as a determinant for restoration of normal gating. Furthermore, they emphasize the relevance of an unaltered structure at the beginning of the *eag* domain sequence to allow its functionally productive interaction with the gating machinery.

### Detection of channel protein and recombinant domain interactions by TIRF/FRET

As a direct test for the physical proximity between the recombinant fragments and the hERG channels showing restored gating, we measured the Förster fluorescence resonance energy transfer (FRET) levels between both putatively interacting partners. For this purpose donor de-quenching following photobleaching of the acceptor fluorophore was used (see Methods), in order to accurately quantify the FRET efficiencies [35]. Measurements were performed under total internal reflection microscopy (TIRF) to selectively measure FRET in the proximity of the plasma membrane, since only fluorescent proteins at a distance of ≈100 nm above the glass coverslip where the cells are attached are studied, thus avoiding most of the contamination from cytoplasmic signals [47]. In our case this could be particularly important because, unlike other typical membrane proteins, only a reduced fraction of the total fluorescence corresponding to properly assembled and fully functional hERG complexes is detected in the perimeter (i.e. the plasma membrane) of the hERG-labeled expressing cells [35]. Furthermore, the TIRF conditions would also tend to minimize the nonspecific signal due to the presence in the cells of the soluble *eag* domain labeled with the acceptor YFP.

**Figure 4:**
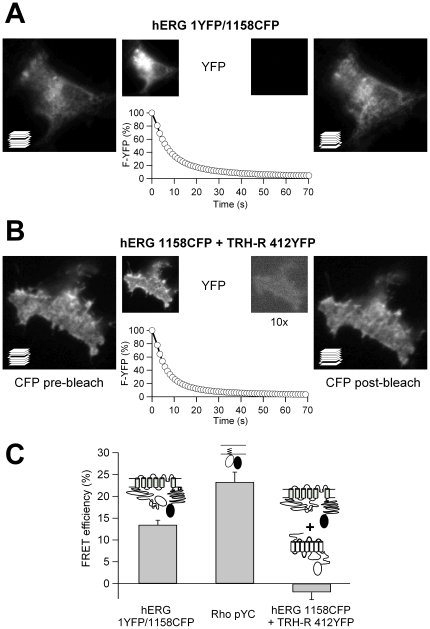
Detection of FRET between hERG N- and C-terminally-located CFP and YFP tags under TIRF conditions, but not between two non-interacting proteins. **A**. Fluorescence micrographs under TIRF illumination of a single cell expressing a hERG construct double-labeled with YFP and CFP in the amino and carboxy terminal ends, respectively. CFP images were obtained before (*left*) and after (*right*) selective photobleaching of YFP as illustrated in the middle panels. CFP and YFP images were adjusted so that the same pixel range was used before and after photobleaching. Note that maximal pixel intensities are not the same for CFP and YFP. Note also the substantial increase in CFP emission after the effective YFP photobleaching, indicative of FRET. A cartoon illustrating the performance of a *z* stack to optimally focus the micrographs corresponding to the CFP emission channel (see Methods) is shown superimposed to the CFP images. **B**. Absence of CFP emission increases in cells co-expressing hERG channels labeled with CFP at the carboxy terminus and TRH receptors C-terminally labeled with YFP. **C**. Positive and negative controls for quantification of E_FRET_ under TIRF microscopy. FRET efficiency was quantified as the fractional increase in CFP emission following photobleaching of YFP as described in Methods. Schematics of the constructs are shown on top of the bars. Data from a hERG construct double-labeled with YFP and CFP at the amino and carboxy terminal ends (hERG 1YFP/1158CFP), a membrane targeted tandem construct of CFP and YFP (Rho-PYC), and co-expressed C-terminally labeled hERG and TRH receptors (hERG 1158CFP+TRH-R 412YFP), are shown. Data from 35, 22 and 15 cells, respectively, were averaged for the graph. White and black ovals represent YFP and CFP, respectively.

The advantages of using TIRF microscopy to optically isolate the fluorescently labeled proteins in the proximity of the plasma membrane, allowing for signal selection from the molecules located there, are illustrated in Fig. S2. Subsequently, we employed the CFP and YFP pairs of labeled channels and recombinant fragments to test for their physical proximity and/or interaction at the plasma membrane using FRET spectroscopy under TIRF conditions. As a positive control, we first used a fully functional hERG construct double-labeled at the amino and carboxy terminal ends (hERG 1YFP/1158CFP), known to yield a substantial level of FRET with epifluorescence imaging [35]. In this case, photobleaching of YFP by more than 90% resulted in a significant increase in CFP fluorescence (Figs. 4A,C), yielding an E_FRET_ value of 13.47±1.04% (n = 35), similar to that obtained with the same construct under wide-field epi-fluorescence (11.64±0.62%, n = 23; p = 0.17, Student's *t* test). The level of FRET was even higher (23.3±2.26%, n = 15) when we used a plasma membrane-targeted fusion construct of CFP and YFP (Rho-pYC) in which a near maximal proximity between both fluorophores is expected (Fig. 4C). By contrast, the averaged FRET efficiency became almost zero when two presumably non-interacting CFP/YFP-tagged proteins were used, namely the hERG channel and the TRH receptor labeled at their carboxy ends (Figs. 4B,C; see also [35]). In all cases, we ensured that no correlation exists between the FRET efficiency of every individual cell and the expression level of the labeled proteins (not shown). These data indicate that the FRET signals are not due to aggregates formed by overexpressed proteins or to random encounters between diffusing fluorophores. Therefore, we used the FRET/TIRF combination as a reporter of proteins in the plasma membrane, to test for the physical proximity between the recombinant *eag* domain and the channel.

**Figure 5:**
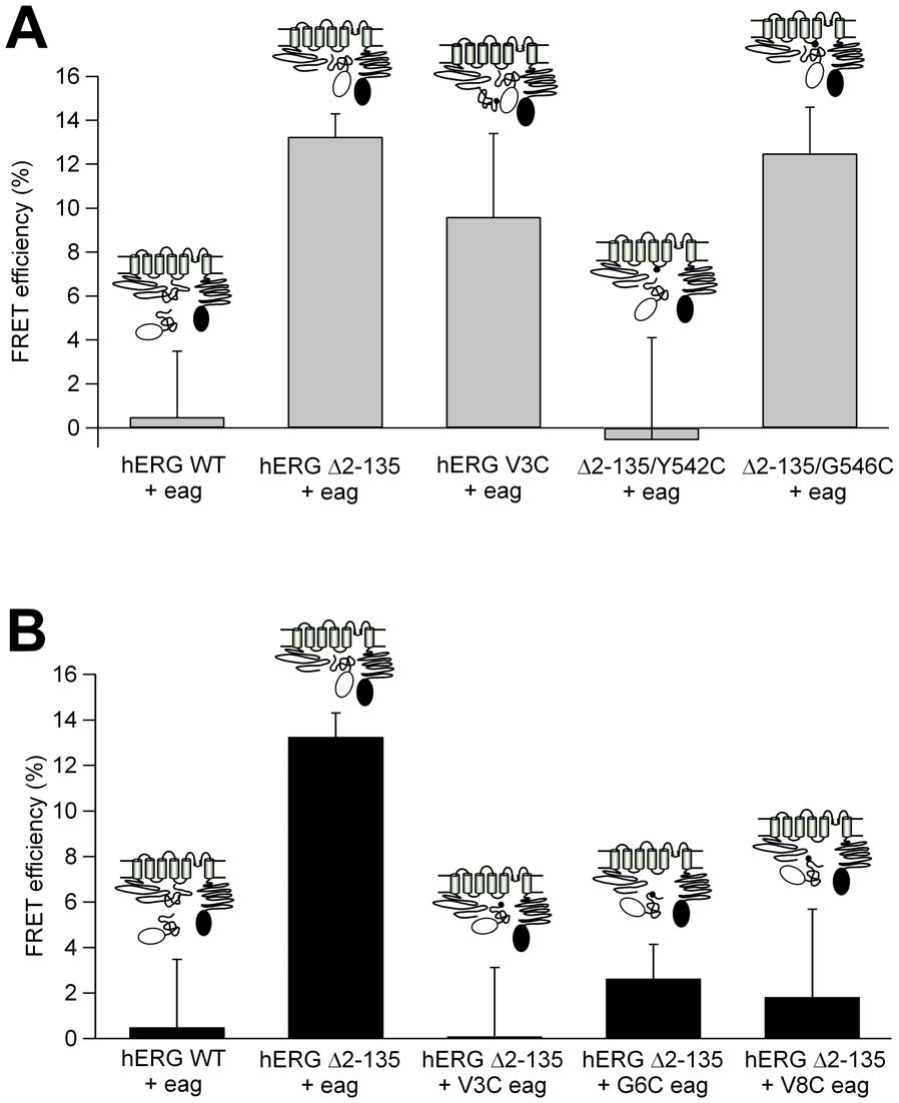
Comparison of FRET/TIRF levels using different hERG constructs and recombinant N1-135 *eag* domain fragments. **A**. Effect of single point mutations in the amino end and the S4–S5 linker of hERG on E_FRET_ levels between recombinant N1-135/YFP *eag* domains and Δ2-135 hERG channels C-terminally labeled with CFP. Averaged data from 7–13 cells are shown. Note the analogous E_FRET_ values obtained with constructs Δ2-135 and 1YFP/1158CFP in panel C of Figure 4, and with hERG Δ2-135/G546C and RhoPYC also in Figure 4C. **B**. Effect of single point mutations in the initial residues of the N1-135/YFP *eag* domain on E_FRET_ levels. Averaged data from 8–13 cells are shown on the graph. Data from WT and Δ2-135 channels co-expressed with recombinant *eag* domains without mutations as in panel A, are also shown for comparison.

In cells coexpressing *eag* hERG domains fused to YFP and hERG Δ2-135 carrying CFP at the C-terminus, the level of E_FRET_ amounted to 13.25±1.05% (n = 12; Fig. 5A). Interestingly, this value was almost the same as that measured with the hERG double labeled at the amino and carboxy ends (see above). This demonstrates that the *eag* domain locates in close proximity to the core of hERG Δ2-135 channels at the membrane. It also suggests that such close proximity may allow for a physical interaction between them related to the functional restoration of normal deactivation by the recombinant *eag* fragment. By contrast, a negligible E_FRET_ was observed between N1-135/YFP and wild-type hERG labeled with CFP at the C-terminus (0.49±2.99%, n = 12). In this case the covalently attached endogenous *eag* domain is probably occluding access of the exogenous domain to its interaction site in the channel core. Finally, a slightly weaker but substantial E_FRET_ (9.61±3.8%, n = 13; *p* = 0.36 *vs* Δ2-135) was observed in cells coexpressing N1-135/YFP and hERG V3C mutant channels C-terminally labeled with CFP.

### Effect of mutations in the S4–S5 linker and the amino end of the *eag* domain on TIRF/FRET levels

We next asked whether the FRET efficiency could be modified by introducing structural perturbations in the putative interacting partners. We observed no FRET when the Δ2-135 channel carried a Y542C mutation at the beginning of the S4–S5 linker (hERG Δ2-135/Y542C, Fig. 5A). By contrast, a FRET signal (12.5±2.1%, n = 11; *p* = 0.66 *vs* Δ2-135) equivalent to that observed with the Δ2-135 channel, was obtained when a cysteine substituted the endogenous glycine in position 546 of this linker (hERG Δ2-135/G546C). As discussed below, these data would be consistent with the low potency of the recombinant fragment to restore the slow deactivation of the Y542C mutant, and also with the full recovery of the slow deactivation kinetics of the Δ2-135/G546C construct. Very reduced E_FRET_ levels were also observed when the recombinant N1-135/YFP fragment carried single point mutations in residues 3, 6 or 8 (V3C, G6C and V8C mutants; Fig. 5B). Thus, the FRET levels were minimized not only in the presence of a mutation that fully knocked out the ability of the recombinant *eag* to restore deactivation kinetics of the hERG Δ2-135 channels (Fig. 3), but also with the V3C and V8C mutated N1-135/YFP fragments, which were able to partially restore hERG Δ2-135 deactivation gating. It seems unlikely that a simple amino acid change in the otherwise unordered initial segment of the *eag* domain [26]–[28], causes a gross perturbation in the general structure of this domain. Therefore, the reduction of FRET in these experiments rather suggests that the mutations could cause a subtle alteration in the relative orientation and/or distance between the fluorophores, due to a weakened interaction of the mutated *eag* domain with the channel. Further work and perhaps use of smaller size and/or differently located fluorophores would be necessary to provide an adequate interpretation of these results.

## Discussion

In this study, we present data indicating that subtle structural variations either in the N-terminal most segment of the *eag* domain, or in the initial portion of the S4–S5 linker, strongly influence the ability of a recombinant *eag* domain fragment to restore normal deactivation gating of hERG channels showing accelerated closing kinetics. Using *Xenopus* oocytes, it has been shown that injection of a peptide corresponding to the entire *eag* domain, direct application of a peptide corresponding to the first 16 amino acids of this domain to excised membrane macropatches, or expression of a recombinant N1-135 *eag* domain, can slow deactivation gating of N-terminally truncated hERG channels [21], [23], [30]. Our results using transfected mammalian cells demonstrate that to achieve complete recovery of normal deactivation properties by the recombinant *eag* domain it is not necessary to delete the whole amino terminus, but only the segment corresponding to this domain. Interestingly, our data also indicate that the exogenously added *eag* domain is able to revert a substantial fraction of the acceleration of closing induced by a single point V3C mutation at the beginning of the hERG amino terminus. The reason for which the recombinant fragment only partially restores slow deactivation of the V3C mutant, even though the mutation is sufficient to cause a similar disabling of the amino terminal-dependent slowing of deactivation as with the more extensive truncations of the N-terminus, remains to be established. It is probable that although the single point mutation is able to cause enough disengagement of the N-terminus from the closing machinery to induce a strong acceleration of closing, some remaining endogenous channel structures may partially antagonize the interaction between the relatively large N1-135/YFP fragment and the V3C channel. This could also be consistent with the fact that the FRET/TIRF signal detected between the recombinant *eag* fragment and the full length hERG V3C/1158CFP channel is slightly smaller than that observed between hERG Δ2-135 and the *eag* domain, which fully restores the slow channel deactivation.

Noticeably, the FRET was negligible between the N1-135/YFP fragment and the Δ2-135 channel carrying the Y542C mutation in the S4–S5 linker, whose deactivation is not slowed down when the recombinant *eag* domain is supplied. In contrast, a maximal level of FRET was obtained with the G546C mutant, in which the slow deactivation rate is fully restored by the exogenous domain. Note, however, that a precise correlation between the quantitative level of FRET between proteins labeled with the quite voluminous GFP-based fluorophores and the extent of the kinetic restoration may be difficult to establish, since the molecular proximity measured by FRET and the specific molecular interaction on which the functional effect relies, may not necessarily coincide [35], [48]. Therefore, although strongly indicative of interactions between the two proteins to which these fluorophores are linked, little indication is provided by the FRET measurements about the short range distances determining the deactivation slowing.

The fact that the *eag* domain-dependent recovery effect is impaired by point mutations both in the initial portions of the N1-135 *eag* domain and in the S4–S5 linker suggests that an interaction takes place between these regions in the full-length channels in order to regulate deactivation gating. This would also be consistent with our recent data showing that a disulfide bond can be formed between the N-terminal most segment of the amino terminus and the initial segment of the S4–S5 linker after placing a pair of engineered cysteines at residues Val3 and Tyr542, respectively [29]. However, whereas the oxidation-dependent modification is preferentially exerted on closed channels locking them in a non-conducting state, the putative interactions proposed here must take place in the open state leading to a slowing of closing. The interaction between the initial segment of the amino terminus and the S4–S5 linker in the closed state of the V3C+Y542C double mutant is fully compatible with the observed modulation of activation properties by the N-terminal-most region of the channel N-terminus and with the reported ability of the Y542C mutation to positively shift the steady-state activation *V*_0.5_ and its less negative ΔG_o_, therefore shifting the equilibrium toward the closed state [25]. Nevertheless, covalently locking the channels in the non-conductive state using disulfide bond formation precludes further analysis of additional interactions and may limit other subsequent dynamic rearrangements of the gating machinery. Thus, it is possible that although subtle differences may exist between the two conformational (and functional) states of the protein, a physical proximity between the amino end and the S4–S5 linker can be maintained in both the closed and the open states. In this context, rather than being considered a static and permanent situation, the *eag*/S4–S5 interaction documented here might be considered as part of a more extensive network of cytoplasmic interactions [29] also involving the PAS and cNBD domains, dynamically modulated during the different phases of the gating process.

The results presented here suggest that the N-terminal most unstructured and flexible segment of the hERG *eag* domain [26]–[28], plays a major role in deactivation slowing through specific interactions with other domains of the channel. This would be consistent with the concept that conformational flexibility and disorder can help some protein domains to engage in fast yet selective interactions crucial for specific functions [49], [50]. Indeed, intrinsic flexibility of the S4–S5 linker, one of the partners of the N-terminus/core interacting pair proposed here, has recently been recognized as an essential modulatory factor of hERG gating [46]. Poor structuralization and flexibility of the N-terminal most segment of the amino terminus has been recognized in *Shaker*-like channels as an important factor for allowing this protein segment to snake its way to reaching its interaction site near the channel and produce N-type inactivation [51]–[53]. If this characteristic is similarly involved in docking the initial segment of the hERG amino end with its interaction site in the channel core (e.g. to the S4–S5 linker) remains an interesting possibility.

It should be noted that our results do not rule out the existence of other sites of interaction between the cytoplasmic domains themselves and between them and the transmembranal core, contributing to modulation of activation and deactivation gating. These could include the intra- and/or inter-subunit interaction of the PAS domain and the amphipathic 13–23 α-helix ahead of it with the C-terminal cNBD [26], [27], [31] and perhaps with some regions of the S4–S5 linker, to adequately position the flexible N-terminal tail region towards the gating machinery [26]. Remarkably, the N-terminal subdomains of the *eag* domain, the PAS domain and the cNBD all appear to be required but none sufficient to maintain slow deactivation kinetics [26], [27], [31]. However, the fact that in the absence of any other structural alteration a subtle change in the N-terminal most segment of the amino terminus (e.g the V3C mutation or the short Δ2–9 deletion [26]) can maximally affect deactivation, points to a direct primary role for this region in the modulation of channel gating.

An interesting observation noted here was that the recovery of normal deactivation kinetics of hERG Δ2-135 channels by the recombinant N1-135 *eag* domain, was not accompanied by a similar reversion of the positive shift in activation voltage dependence caused by the deletion. This suggests that the influence of the amino terminus on activation is exerted by a different mechanism that remains impaired in the presence of the recombinant fragment. Obvious candidates to participate in this phenomenon are the N-terminus proximal domain, the S4–S5 linker and the C-terminus C-linker/CNBD. In the first possibility, alterations in the electrostatic influence exerted by an amino acid cluster near the S1 transmembranal segment on the gating machinery at the level of the S4–S5 linker [24], [40] could be involved. Interestingly, it has been suggested that this influence may depend on the proper orientation of the first 16 N-terminal residues of the *eag* domain toward the S4–S5 linker in the channel core [25]. Note, however, that some hormonal effects on activation gating that require an intact amino terminus, are exerted in the absence of this cluster [34]. Finally, a significant impact of the CNBD on hERG activation, perhaps independent of any interaction with the PAS domain and apart from its influence on deactivation gating, has also recently been described [31].

In summary, based in our work and that of others [26]–[31] we propose an unifying model, in which the PAS domain and the amphipathic α-helix ahead of it act as a scaffold and a spacer helping to correctly orientate the initial unstructured and flexible tail of the amino end towards the amino terminal portion of the S4–S5 linker to modulate hERG gating (this report and [26], [29]). As described for the C-terminal C-linker/cNBD regions in HCN2 [54], these regions of hERG could hang centrally below the transmembrane core, establishing at their top and side surfaces extensive contacts with the more peripheral *eag* domains [35]. This would help to place the PAS domain and the amphipatic α-helix in position allowing them to also influence channel gating, via the S4–S5 linker and/or allosterically through their interaction with those C-terminal structures [27], [31] that are directly linked to the channel gate at the bottom of helix S6. Altogether, this would strengthen our recent proposal that physical interactions between some cytoplasmic hERG domains constitute an essential component of the gating machinery [29]. Further work will be necessary to confirm the overall validity of our model and, in particular, to unravel the details about its conformational rearrangements and dynamic variations during the functionality of the channel.

## Supporting Information

Figure S1 **Recombinant N1-135** ***eag*** **domain fragment coexpression has no effect on activation voltage dependence rightward shifts caused by the Δ2-135 deletion.** Pulse protocol and voltage-clamp recordings of a family of currents from h-ERG Δ2-135 channels in the absence or the presence of coexpressed N1-135/YFP recombinant *eag* domain fragments, are shown at the *top*. Normalized *I/V* relationships for the indicated constructs are shown at the *bottom*. Fractional activation curves were obtained from tail current data at −50 mV after 1 or 5 s depolarizations between −80 and +80 mV in 20 mV increments from a holding potential of −80 mV. The continuous lines correspond to Boltzmann curves *h*(*V*) = *I_max_* [1/(1+exp((*V−V_0.5_*)/*k*))], which best fitted the data. Plots corresponding to wild-type hERG with and without CFP label in the carboxy end (ref. 35) are also shown for comparison.
(TIF)

Figure S2 **Selective excitation of fluorophores at plasma membrane and submembrane regions near the coverslip-cell interface under TIRF illumination.** **A**. Comparison of cell fluorescence images under wide-field epi-fluorescence (*left*) and TIRF illumination (*right*) after focusing the objective at the level of the glass coverslip-water interface. Note the notorious reduction of the background and out-of-focus fluorescence under TIRF conditions, associated with a remarkable increase in the sharpness and contrast of the layer corresponding to the cell footprint in contact with the glass, mainly representing the plasma membrane environment. Fluorescence micrographs correspond to a HEK-293 cell expressing YFP-labeled TRH receptors known to preferentially distribute in the plasma membrane (ref. 35). **B**. TIRF-induced preferential photobleaching of labeled proteins in and near the plasma membrane abutting the coverglass. Note the very similar fluorescence levels observed in the cells imaged with epifluorescence microscopy and focused near the cell center, both before and after nearly complete selective photobleaching with TIRF illumination of the layer in contact with the glass surface. This indicates that TIRF-based photobleaching preferentially targets fluorescence emissions from YFP-labeled TRH-R molecules at/near the plasma membrane compared with other cellular pools of the same molecules.
(TIF)

Table S1 **Comparison of activation parameters for all constructs in the presence and the absence of the recombinant fragment.**
(DOC)
