# Supplementary figures and images for: Molecular Determinants of Interactions between the N-Terminal Domain and the Transmembrane Core That Modulate hERG K+ Channel Gating
Source: PLoS One. 2011 Sep 15;6(9):e24674. doi: 10.1371/journal.pone.0024674 (PMC3174182; doi:10.1371/journal.pone.0024674)

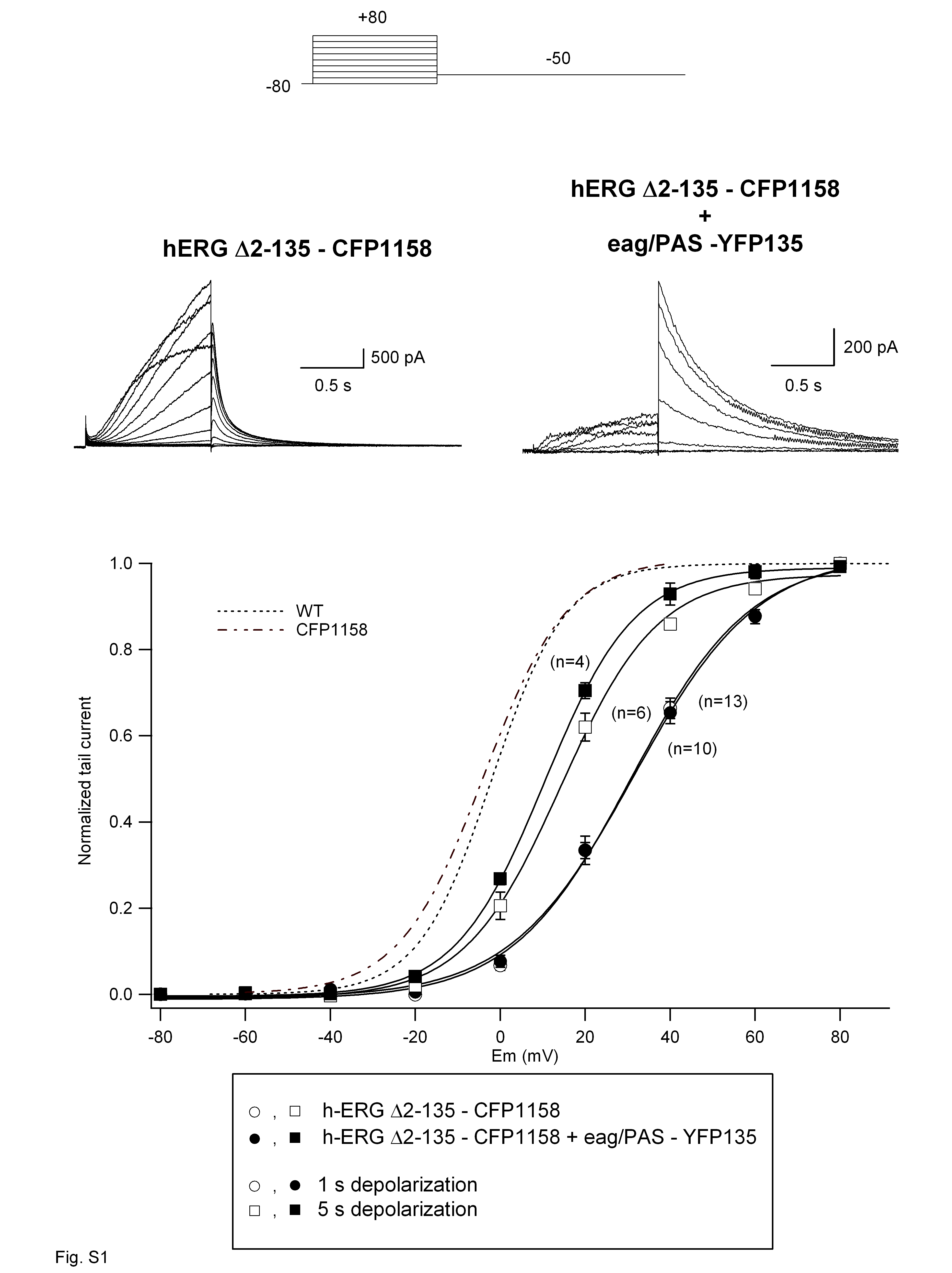

Supplement: Figure S1 — Recombinant N1-135 eag domain fragment coexpression has no effect on activation voltage dependence rightward shifts caused by the Δ2-135 deletion. Pulse protocol and voltage-clamp recordings of a family of currents from h-ERG Δ2-135 channels in the absence or the presence of coexpressed N1-135/YFP recombinant eag domain fragments, are shown at the top. Normalized I/V relationships for the indicated constructs are shown at the bottom. Fractional activation curves were obtained from tail current data at −50 mV after 1 or 5 s depolarizations between −80 and +80 mV in 20 mV increments from a holding potential of −80 mV. The continuous lines correspond to Boltzmann curves h(V) = Imax [1/(1+exp((V−V0.5)/k))], which best fitted the data. Plots corresponding to wild-type hERG with and without CFP label in the carboxy end (ref. 35) are also shown for comparison. (TIF) [file pone.0024674.s001.tif]

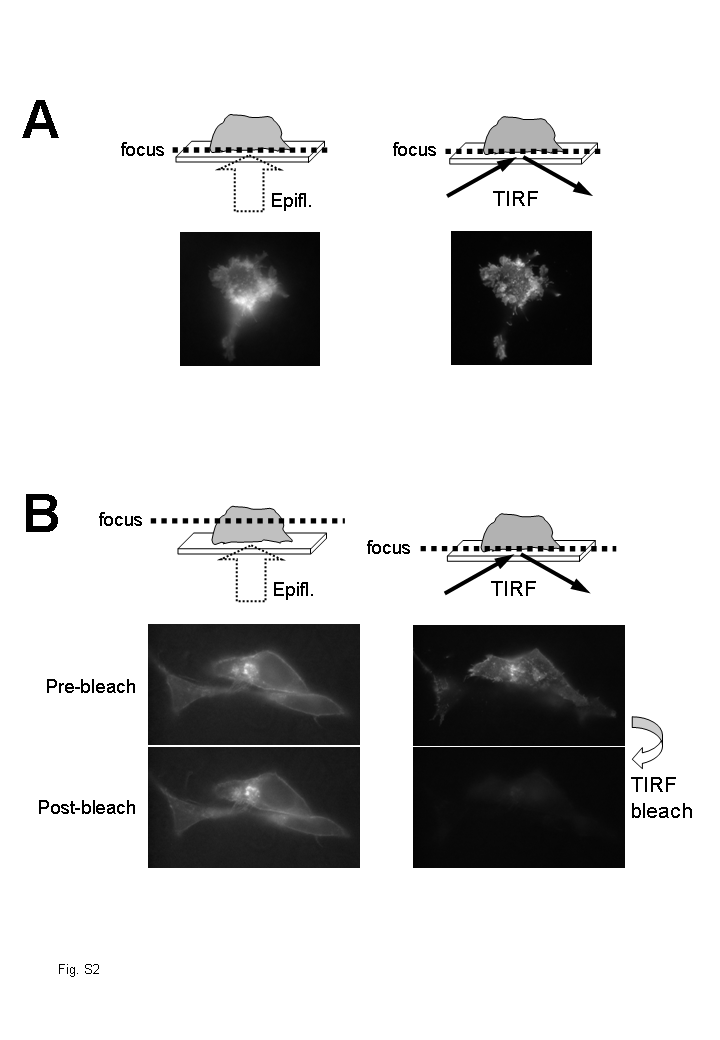

Supplement: Figure S2 — Selective excitation of fluorophores at plasma membrane and submembrane regions near the coverslip-cell interface under TIRF illumination. A. Comparison of cell fluorescence images under wide-field epi-fluorescence (left) and TIRF illumination (right) after focusing the objective at the level of the glass coverslip-water interface. Note the notorious reduction of the background and out-of-focus fluorescence under TIRF conditions, associated with a remarkable increase in the sharpness and contrast of the layer corresponding to the cell footprint in contact with the glass, mainly representing the plasma membrane environment. Fluorescence micrographs correspond to a HEK-293 cell expressing YFP-labeled TRH receptors known to preferentially distribute in the plasma membrane (ref. 35). B. TIRF-induced preferential photobleaching of labeled proteins in and near the plasma membrane abutting the coverglass. Note the very similar fluorescence levels observed in the cells imaged with epifluorescence microscopy and focused near the cell center, both before and after nearly complete selective photobleaching with TIRF illumination of the layer in contact with the glass surface. This indicates that TIRF-based photobleaching preferentially targets fluorescence emissions from YFP-labeled TRH-R molecules at/near the plasma membrane compared with other cellular pools of the same molecules. (TIF) [file pone.0024674.s002.tif]
