# Supplementary material for: Molecular Determinants of Interactions between the N-Terminal Domain and the Transmembrane Core That Modulate hERG K+ Channel Gating
Source: PLoS One. 2011 Sep 15;6(9):e24674. doi: 10.1371/journal.pone.0024674 (PMC3174182; doi:10.1371/journal.pone.0024674)
Supplement: Table S1 — Comparison of activation parameters for all constructs in the presence and the absence of the recombinant fragment. (DOC) [file pone.0024674.s003.doc]

**Table S1. Comparison of activation parameters for all constructs in the presence and the absence of the recombinant fragment.**

**Construct V_0.5_ (mV) *k* n**

∆2-370 18.9 ± 5.1 12.6 ± 0.90 5

∆2-370 + eag fragment 16.8 ± 1.8 14.0 ± 1.00 6

∆2-135 29.9 ± 2.1 12.4 ± 0.42 14

∆2-135 + eag fragment 28.9 + 1.5 13.9 + 0.60 16

V3C 24.4 ± 3.9 12.2 ± 0.77 10

V3C + eag fragment 30.2 ± 2.5 11.4 ± 0.51 5

Y542C 38.4 ± 0.5 10.0 ± 0.75 3

Y542C + eag fragment 41.2 ± 3.4 11.9 ± 0.60 4

∆2-135 /Y542C 31.3 ± 2.9 15.1 ± 0.10 3

∆2-135/Y542C + eag fragment 35.4 ± 4.6 15.7 ± 1.50 3

∆2-135 /G546C 8.5 ± 1.1 14.2 ± 0.43 7

∆2-135/G546C + eag fragment 15.9 ± 3.2 17.0 ± 0.64 8

∆2-135 + V3Ceag 40.1 ± 5.8 13.5 ± 2.40 4

∆2-135 + R4Ceag 31.5 ± 4.1 14.1 ± 1.00 6

∆2-135 + G6Ceag 28.4 ± 2.9 10.3 ± 0.44 10

∆2-135 + V8Ceag 31.3 ± 3.4 15.0 ± 1.50 10

V_0.5_ and *k* values were obtained as indicated in the legend of supplementary figure 1 from Boltzmann curves of the type *h*(*V*) *= I_max_* [1/(1 + exp((*V – V_0.5_*)/*k*))] following 1 s depolarizations. Statistically significant changes in the values of the kinetic parameters are not observed in the presence of the recombinant fragment for any of the tested constructs.
